# “HER2-Low” and the Challenge of Repurposing Legacy HER2 IHC Biomarker Assays

**DOI:** 10.3390/curroncol33040181

**Published:** 2026-03-25

**Authors:** Emina E. Torlakovic, Allen M. Gown

**Affiliations:** 1 Department of Pathology and Laboratory Medicine, University of Saskatchewan and Saskatchewan Health Authority, Saskatoon, SK S7N 0W8, Canada; 2 Department of Pathology, University of British Columbia, Vancouver, BC V6S 0M4, Canada

**Keywords:** HER2-low, biomarker repurposing, immunohistochemistry, trastuzumab deruxtecan, companion diagnostic, assay validation, breast cancer

## Abstract

In September 2022, the US Food and Drug Administration (FDA) expanded approval of the legacy Roche monoclonal antibody 4B5-based immunohistochemistry (IHC) assay to identify patients with HER2-low breast cancers predicted to respond to trastuzumab deruxtecan (T-DXd), based on findings from the DESTINY-Breast04 and DESTINY-Breast06 clinical trials. However, this repurposing of a legacy biomarker assay raises significant clinical and technical validation concerns. The legacy HER2 IHC assay was originally developed to identify tumors with HER2 overexpression (3+) resulting from gene amplification, which leads to substantially higher HER2 receptor expression than in low/ultralow expression tumors. The current application to distinguish HER2-low and HER2-ultralow tumors from truly HER2-negative tumors represents a fundamentally different biological and clinical purpose, yet the assay’s analytical and clinical validation for this new purpose remains incomplete. Critical gaps include the lack of established analytical sensitivity and specificity for identifying 1+ HER2 cases, poor reproducibility of pathologist scoring at low HER2 levels, and the absence of alternative methodologies for orthogonal validation. While more sensitive quantitative approaches (such as AQUA) may detect additional low HER2 expression cases missed by conventional IHC, increased analytical sensitivity does not automatically translate to clinical utility. Furthermore, the ASCO/CAP guidelines cutoffs were developed for HER2 overexpression detection and may not be the best choice for HER2-low/ultralow identification without clinical validation and determination of fit-for-purpose analytical specifications. We examine the current challenges of repurposing legacy HER2 IHC biomarker assays for HER2-low detection, evaluating the precedent of other repurposed IHC assays (ALK, CD30, and PD-L1), and emphasizing the necessity for proper technical and clinical validation before widespread implementation. We conclude that prospective clinical trials are essential to establish clinically meaningful cutoffs and analytical specifications appropriate for patient selection in HER2-low disease.

## Simple Summary

This is a critical review of a “repurposed” laboratory assay originally designed for the detection of the breast cancer protein HER2, which is overexpressed on the surface membrane of a subset of breast cancer tumors. In its original use, this immunohistochemical (IHC) test, which identifies positive tumors by allowing visualization of membrane staining on a glass slide examined by a pathologist, was able to identify the subset of breast cancer patients with a significant likelihood of responding to a targeted therapy, such as trastuzumab. More recently, patients with much lower levels of HER2 protein expression have been shown to respond to a modified form of targeted therapy, in which trastuzumab is coupled to deruxtecan, a cytotoxic molecule. The questions discussed include whether the original IHC test can be reliably applied to tumors with this lower level of HER2 expression with appropriate sensitivity and specificity and whether the assay has been correctly validated in both the technical and clinical realms for this new purpose.

## 1. Introduction

In September 2022, the US Food and Drug Administration (FDA) expanded the prior approval of the Roche monoclonal antibody 4B5-based immunohistochemistry (IHC) assay, which had previously been approved to identify patients with 3+ HER2 expression in their breast cancers [1]. In the new approval, this legacy IHC-based HER2 assay was deemed capable of identifying patients whose tumors showed “HER2-low”, which strongly overlaps with those cases previously called 1+ HER2 (and deemed negative for overexpression). This recommendation was based on the results of the DESTINY-Breast04 clinical trial, in which a favorable patient response to the antibody drug conjugate (ADC), T-DXd, was demonstrated [2]. The efficacy of T-DXd was confirmed in the DESTINY-Breast06 clinical trial, which showed that significant responses were also seen in patients whose tumors showed any positivity for HER2 protein expression [2]. These studies compared the efficacy of second-line T-DXd vs. chemotherapy in patients with tumors that did not manifest strong HER2 overexpression but were not designed to test the ability of the legacy HER2 IHC assay to identify patient responders. Indeed, only patients showing weak HER2 positivity were included in the studies (no patients with HER2 scores of 0 were included). While the FDA recognized that this nevertheless represented a new “purpose” for the HER2 IHC assay, i.e., to now identify low-level HER2 expression, it follows that separate approval for this purpose was required. Although the clinical trials demonstrated that this HER2 IHC assay can indeed identify a population of patients who will respond to the new therapy, there were several omissions in the documentation regarding the applicability of this legacy IHC assay to this new purpose. These omissions, together with other data on HER2 IHC assay performance, raise serious questions about the less-than-perfect validity of the application of this legacy HER2 IHC assay to this new implied purpose.

## 2. What Is IHC Assay Repurposing?

While there is a long history of repurposing IHC biomarkers, this has, unfortunately, most frequently been done without any special considerations for the clinical and technical validation necessary for the new purposes. Examples of previously repurposed IHC biomarker assays appear in the accompanying table and include: ALK IHC, an IHC assay originally meant for the accurate diagnosis of ALCL, repurposed for the detection of ALK fusions in lung cancer [3]; CD30 IHC assays, used for the diagnosis of CD30-positive hematological and other tumors, repurposed for use in stratifying patients with T-cell lymphoma for targeted therapy with brentuximab vedotin [4]; and Ki-67 IHC assays, initially developed as diagnostic biomarkers, now used as prognostic biomarkers in the context of high-risk early breast cancer [5,6]. There has also been repurposing of one PD-L1 predictive biomarker assay for another indication (e.g., PD-L1 IHC, developed for pembrolizumab therapy eligibility in non-small cell lung cancer, repurposed for use in gastric cancer to be treated with nivolumab [7,8]). Table 1 summarizes the background of this IHC assay repurposing.

## 3. HER2 Assays Identify Biological Differences Between Tumors with HER2 Overexpression (“Positive”) and Tumors Without HER2 Overexpression (“Negative”)—Differences That Predict Response to Trastuzumab

**Figure 1:**
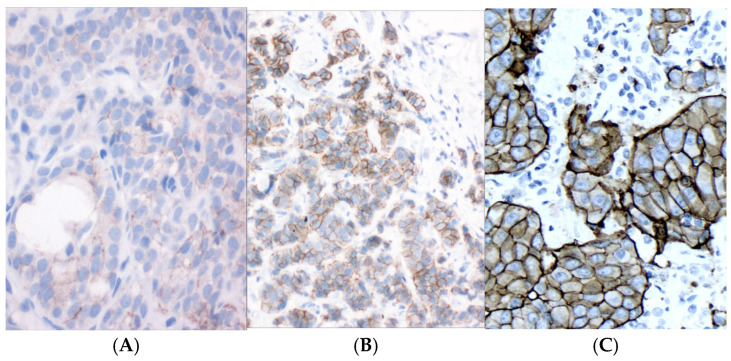
HER2 immunohistochemistry showing differences among 1+ (**A**), 2+ (**B**), and 3+ (**C**) tumors.

The “legacy” HER2 assays, such as Herceptest and the monoclonal antibody 4B5-based assays, had the purpose of detecting overexpression of HER2, which, in almost all cases, is a direct result of HER2 gene amplification [9]. Examples of the three levels of positive HER2 expression (1+, 2+, and 3+) are shown in Figure 1A–C.

As a consequence, breast cancers showing gene amplification (and 3+ HER2 signal) manifest between 100,000 and 2,000,000 HER2 receptors per tumor cell, whereas breast cancers without gene amplification (e.g., 0 and 1+ using legacy assays) express fewer than 100,000 HER2 receptors per cell [10,11]. HER2 2+ tumors are considered indeterminate and require FISH studies to determine HER2 status and, hence, eligibility for treatment with trastuzumab. It has been documented that there are significant quantifiable differences in HER2 protein expression in amplified/overexpressed (3+) versus HER2-negative breast cancers using these legacy assays, and these biological differences are reflected in the clinical response to trastuzumab, manifesting only in a subset of the 3+/overexpressed group [9]. Importantly, the terminology applied to the pathologist’s readout (scoring the tumors as 3+ or 2+ and FISH/CISH-positive) was interpreted in this context as tumors “positive” for HER2, although it was clear that many tumors without amplification also showed some positivity for HER2 expression, albeit below the levels required to identify HER2 overexpression.

## 4. In Contrast, Legacy HER2 Assays Are Not Known to Highlight Biological Differences Between HER2-Low and/or HER2-Ultralow and Truly Negative Tumors, and There Are No Data to Determine Whether Identifying Breast Cancers with HER2-Low/Ultralow Can Separate Breast Cancer Patients into Those Predicted to Respond to T-DXd and Those Not Predicted to Respond

While there are differences in documented HER2 expression in IHC 0 (none to up to 10,000 molecules per cell). vs. IHC 1+ (10,000 to 100,000 molecules per tumor cell) breast cancers [10,11], and there is evidence that patients with breast cancer without HER2 gene amplification or overexpression can respond to T-DXd, because there is currently no evidence that HER2 IHC can distinguish between responders and non-responders to T-DXd. In the DESTINY 04 and 06 clinical trials, where the PATHWAY anti-HER2/neu (4B5) antibody assay was used, no patients completely negative for HER2 were included; hence, the response rate in this group is unknown. Importantly, there should be no terminological confusion between the scoring and the tumors being interpreted as “positive” or “negative”. At present, HER2 “zero” is a scoring category that includes tumors that are truly negative and those with very low expression of HER2 that do not qualify as 1+ according to ASCO/CAP scoring criteria [12]. However, with the new purpose, ASCO/CAP scoring should not be applied at all, as it was developed to distinguish tumors with overexpression from those without overexpression. Once the tumors without overexpression are identified, scoring relevant to the new purpose should be applied and reported specifically for that new purpose. In this context, once tumors with HER2 overexpression are excluded, it is more appropriate to classify the remaining tumors as HER2-negative or HER2-low/ultralow.

In addition to questionable biological differences between HER2-low/ultralow and HER2-negative 1+ and HER2 0 breast cancers, pathologists’ classification of 0 and 1+ is highly irreproducible, as discussed below. However, data on pathologist accuracy and precision using a more relevant scoring system for the new purpose (HER2-negative vs. HER2-low/ultralow) are less explored.

Another challenge in repurposing legacy HER2 IHC assays is that there are currently no alternative methodologies to help distinguish 0 and 1+ HER2 tumors from one another, or even HER2-negative from HER2-low/ultralow. In contrast, in the case of the legacy HER2 IHC assay (performed to identify patients who may respond to trastuzumab), fluorescence or chromogenic in situ hybridization, looking directly at the tumor nuclei for amplification of HER2, can serve as an excellent alternative method for identification of the 3+ group, as well as a means for HER2 IHC assay validation.

## 5. When and How Should Legacy IHC Biomarkers Be Repurposed?

It is only recently that the “fit-for-purpose” approach has been introduced to IHC and its importance emphasized for the validation of IHC assays [13]. Diagnostic laboratories must consider the purpose of a biomarker assay before introducing it into clinical practice. If pathologists or oncologists decide to use an established biomarker assay for a new purpose, this becomes a new biomarker assay, irrespective of the fact that the technical specifications of the protocol did not change. To use an existing biomarker assay for a new purpose requires new validation because biomarker assays are defined by their purpose. This also includes a consideration of applying a different readout/scoring system that is potentially more suitable for the new purpose.

One of the lessons learned from ALK IHC assay repurposing is that consideration of the technical performance of an IHC biomarker protocol, most importantly its analytical sensitivity and the required new clinically relevant low limit of detection (LOD), as well as pathologist readout accuracy for IHC assays, may be considerably different for different applications, purposes, or uses [13,14]. Even a suboptimal ALK IHC assay protocol with an inappropriate LOD may detect a sufficient number of lung tumors with ALK rearrangements to show good correlation with FISH, which on its own could reach statistical significance. However, the legacy ALK IHC assay missed about 40% of patients who otherwise qualify for therapy with legacy ALK inhibitors [3].

Unfortunately, we have no direct evidence about the LOD or the sensitivity and specificity of the legacy HER2 IHC assay in identifying 1+ HER2 cases, given the absence of a “ground truth”, i.e., an independent assessment of the levels of HER2 expression. The development of assays that can quantitatively determine the number of HER2 receptors on the tumor cell surface, or an accurate surrogate, could help in this regard. The recently described CASI-01 IHC reference standards were demonstrated to be capable of measuring the analytical sensitivity of different HER2 IHC assays, but these calibrators cannot measure actual levels of expression in tissue. An HER2 IHC assay would need to be developed as a quantitative assay in order to do so, which is not possible at this time [15]. In addition, there is AQUA (Automated Quantitative Analysis), which is an image-analysis-based method for measuring in situ protein expression on tissue sections using fluorescence signals rather than chromogenic 3,3′-diaminobenzidine (DAB), the standard brown stain used in IHC protocols. This assay can report quantitative measurements of HER2 expression in tumors [16,17]. Either of these approaches could conceivably be applied in order to validate IHC assays for diagnostic accuracy in HER2-low/ultralow vs. HER2-negative breast tumors. Importantly, this does not automatically mean that more analytically sensitive assays would have better diagnostic sensitivity and specificity. It is only after this is assessed in clinical trials that we will be able to tell what analytical sensitivity is required to determine clinically relevant levels of expression for this new purpose, i.e., the prediction of response to T-DXd.

Unfortunately, at present, there are no clear guidelines for clinical laboratories on how to manage the revalidation of repurposed biomarkers, not only for HER2-low/ultralow but also for other biomarkers. However, it is clear that repurposing legacy IHC assays requires both technical/analytical and clinical or at least indirect clinical validation [18].

## 6. Technical Validation

Technical validation requires determination of analytical sensitivity, specificity, reproducibility, reportable range, technical accuracy, and pre-analytical robustness [19]. It has long been noted that pre-analytic parameters can result in poor performance of legacy HER2 IHC assays, but this has been in large part obviated by the publication of ASCO-CAP guidelines, which prescribe laboratory practices to standardize pre-analytic parameters, such as fixation type and duration [12].

Uniformity of sensitivity in HER2 IHC assays across various laboratories and tissue sources is one of the critical objectives of technical validation. This was highlighted in the study by Gown et al. almost twenty years ago [20], when it was demonstrated that a “normalization” of the HER2 signal on the tumor (i.e., subtracting the score of the tumor from the apparent score of the normal breast epithelium, presumably correcting for differences in sensitivity in tissues fixed and processed in different laboratories) was required to achieve high levels (94.7%) of concordance between HER2 assessment by IHC and by FISH [20] in specimens from many different sources.

When an IHC assay is not set up to have the best analytical sensitivity and specificity, the readout/scoring system and the cutoff may not be the most clinically meaningful and may not be the most discriminative in selecting the patients for appropriate therapies. Therefore, clinical trials should not select already available IHC assays without addressing this critical issue. The processes and procedures required to establish IHC assays with the best analytical sensitivity and specificity are known and currently accomplished in the context of proficiency testing programs for other IHC assays (e.g., keratin, S-100, CD45, etc.).

Technical accuracy of the HER2 IHC assay can, in the case of legacy HER2 IHC testing for overexpression of HER2 protein, be determined by comparison with an alternative testing methodology (e.g., FISH or CISH). Because there is no widely available alternative testing modality for the determination of “HER2-low”, the precision of this repurposed assay is indeterminate. However, at least for the purpose of clinical trials, AQUA methodology and BCS calibrators could be utilized.

In a number of recently published studies, only moderate to poor reproducibility of the legacy HER2 IHC assays in the context of identifying HER2-low breast tumors has been demonstrated, although the rate of discordance can be somewhat reduced with pathologist training [21,22,23,24,25,26,27,28,29,30,31]. However, we do not have data on the other components of the technical validation. Specifically, since a major component of the technical aspect of any IHC assay requiring validation is the reproducibility of the readout, and given the challenges in the reproducibility of HER2-low (1+) IHC, this may be problematic for the repurposing of the legacy HER2 IHC assay. However, the recent CASI study [15] and a few others have shown that artificial intelligence-based algorithms may be more reliable than the human eye at this low threshold [32,33].

## 7. Clinical Validation

The DESTINY-Breast04 and 06 studies did provide direct clinical validation for T-DXd efficacy in HER2-low/ultralow tumors as detected by the PATHWAY anti-HER2/neu antibody assay, as these were prospective randomized trials. Patients were enrolled based on local HER2 IHC scoring (as well as negative FISH status in cases of HER2 IHC 2+). These studies unequivocally demonstrated the superiority of T-DXd versus the physician’s choice of chemotherapy [2].

As discussed above, since the clinical trials used the legacy HER2 assay with unknown performance characteristics for this new purpose, and based on recently published evidence using AQUA methodology, it is clear that the current gold standard does not identify all HER2 molecules in breast cancer. Therefore, it is not known whether the current gold standard is an optimal assay possessing the accuracy (high diagnostic sensitivity and specificity) to identify the largest number of patients with a chance of responding to the new therapy and to exclude patients who have no chance or a negligible chance of response. Maximizing specificity is also important because ADC agents such as T-DXd may have significant toxicity and side effects; therefore, it is possible that the current gold standard may indeed be better than more sensitive assays, assuming that the readout/scoring is adjusted to overall analytical sensitivity.

## 8. Conclusions

In approving the Roche 4B5 antibody-based legacy HER2 IHC assays for a new and different purpose, i.e., the determination of “HER2-low” expressing breast tumors, the FDA has, in fact, repurposed this legacy assay for the distinction of levels of HER2 expression that do not correspond to known changes in gene expression or for which there are currently no widely available alternative methodologies for accurate classification. In fact, the validation gleaned from the published studies [2] shows only that a subset of patients with HER2-low/ultralow tumors responded to T-DXd, having better clinical outcomes than patients treated with chemotherapy, but it is unknown from these published studies or the FDA approval based on them whether this assay can predict an absence of response in patients with HER2-negative tumors.

It may well be that legacy HER2 IHC assays can be repurposed as proposed, but new studies are showing that more sensitive assays are detecting more breast cancers as “positive for low HER2 expression” and are challenging the use and the approval of the legacy assay. The only way to know if more sensitive assays are better at distinguishing between responders and non-responders is to use those sensitive assays in clinical trials. However, such evidence is not yet available, and the assays that are currently employed in this context may pose a potential risk to the clinical team treating breast cancer patients. That said, we should not stop issuing results for HER2-low/ultralow using the PATHWAY anti-HER2/neu (4B5) antibody and/or LDTs that show nearly identical performance characteristics in indirect clinical validation because there is clear evidence that this group of patients can benefit from this treatment. The question remains whether some of the patients who are identified as “negative for HER2” for this purpose may qualify for treatment if IHC assays with higher analytical sensitivity are used. Alternatively, there is a possibility that further analysis will demonstrate that even HER2-negative patients show clinical response. This could mean that there are no “true negative” HER2-expressing tumors, potentially obviating the rationale for HER2 testing.

## Figures and Tables

**Table 1:** Examples of repurposed IHC biomarker assays in oncology.

Biomarker	Methodology	Original Purpose	New Purpose
ALK	IHC (LDT *, ALK1 clone)	Diagnostic for anaplastic large cell lymphoma	Predictive of response to ALK inhibitors in non-small cell lung cancer
CD30	IHC (LDTs *)	Diagnostic for Hodgkin’s lymphoma and anaplastic large cell lymphoma	Predictive of response to brentuximab vedotin in T-cell lymphoma
PD-L1	IHC (22C3 PharmDx)	Predictive of response to first-line pembrolizumab in NSCLC	Predictive of response to nivolumab in upper GI adenocarcinoma
HER2	IHC companion diagnostic and LDTs, *	Predictive of response to HER2-directed monoclonal antibody therapy in breast cancer with amplified/overexpressed HER2	Predictive of treatment response to T-DXd in HER2-low breast cancer

* LDT = laboratory-developed test.

## Data Availability

No new data were created or analyzed in this study.
